# Fluorinated Multi-Walled Carbon Nanotubes Coated Separator Mitigates Polysulfide Shuttle in Lithium-Sulfur Batteries

**DOI:** 10.3390/ma16051804

**Published:** 2023-02-22

**Authors:** Devashish Salpekar, Changxin Dong, Eliezer F. Oliveira, Valery N. Khabashesku, Guanhui Gao, Ved Ojha, Robert Vajtai, Douglas S. Galvao, Ganguli Babu, Pulickel M. Ajayan

**Affiliations:** 1 Department of Material Science and NanoEngineering, Rice University, Houston, TX 77005, USA; 2 Group of Organic Solids and New Materials, Gleb Wataghin Institute of Physics, University of Campinas (UNICAMP), Campinas 13083-970, SP, Brazil; 3 Center for Computational Engineering & Sciences (CCES), University of Campinas (UNICAMP), Campinas 13083-970, SP, Brazil

**Keywords:** lithium-sulfur batteries, energy storage, fluorinated multi-walled carbon nanotubes, interlayer, polysulfide shuttle

## Abstract

Li-S batteries still suffer from two of the major challenges: polysulfide shuttle and low inherent conductivity of sulfur. Here, we report a facile way to develop a bifunctional separator coated with fluorinated multiwalled carbon nanotubes. Mild fluorination does not affect the inherent graphitic structure of carbon nanotubes as shown by transmission electron microscopy. Fluorinated carbon nanotubes show an improved capacity retention by trapping/repelling lithium polysulfides at the cathode, while simultaneously acting as the “second current collector”. Moreover, reduced charge-transfer resistance and enhanced electrochemical performance at the cathode-separator interface result in a high gravimetric capacity of around 670 mAh g^−1^ at 4C. Unique chemical interactions between fluorine and carbon at the separator and the polysulfides, studied using DFT calculations, establish a new direction of utilizing highly electronegative fluorine moieties and absorption-based porous carbons for mitigation of polysulfide shuttle in Li-S batteries.

## 1. Introduction

Lithium-sulfur battery (Li-S) has become a promising candidate as next generation rechargeable energy storage device due to a high theoretical capacity of sulfur (1675 mAh g^−1^). Sulfur is cheap, environmentally benign, and delivers high gravimetric energy density, making it an exceptional alternative to expensive and toxic metal oxide-based cathode materials [1]. However, commercialization of Li-S batteries is still hampered by two major challenges: low electrical conductivity of sulfur, and polysulfide (PS) shuttle [2]. During the discharge process of a S cathode, an α-sulfur (S_8_) molecule accepts two electrons by opening its puckered ring structure to form Li_2_S_8_. Each “round” of Li addition involves adding up a pair of electrons creating intermediate redox species until the formation of Li_2_S as the final discharge product. These intermediate species, commonly known as polysulfides (Li_2_S_n_, 2 < n ≤ 8), are soluble in commonly used ether-based electrolytes [3,4]. A shuttle cycle occurs when the higher order polysulfides (Li_2_S_n_, 4 ≤ n ≤ 8), formed due to the initial reduction of the sulfur cathode, migrate towards the anode to form lower order polysulfides (Li_2_S_n_, 2 < n ≤ 4) and return back to the cathode to re-form higher order polysulfides [5,6]. This undesirable migration promotes a significant decline in coulombic efficiency, and can lead to the formation of an insulating and insoluble Li_2_S layer at the anode resulting in an irreversible capacity fade [5].

Past research works on improving the cycle life of Li-S batteries have mainly concentrated on cathode [4,7,8,9,10], anode [11,12], and electrolyte [13,14,15,16]. Separator, an indispensable component of current Li-secondary batteries, is an insulating permeable membrane that allows the transport of solvated species between cathode and anode. Recently, there has been growing interest in developing “bifunctional separator” membranes to alleviate PS shuttle [6,17,18,19,20,21,22]. One of the common strategies is to incorporate polar materials on the separator. Babu et al. incorporated hexagonal boron nitride on the surface of a polypropylene Celgard separator using a facile vacuum filtration process and suggested that the adsorption of polysulfides at B-N sites occurred via Lewis acid-base interaction [23]. Yao et al. deposited metal oxides onto the surface of a Celgard separator; however, a low electronic conductivity of these materials can lead to incomplete utilization of sulfur at the cathode [24]. Contrarily, carbon-based materials with good natural electrical conductivity, such as mesoporous carbon [25,26], MWCNTs [26,27], and microporous carbon paper, [28,29] reduce this issue by functioning as a “second current collector” and as a trap for polysulfides [26]. Moreover, these lightweight carbon-based materials have a high surface area to volume ratio, which improves the sulfur utilization and results in a minimum impact on volumetric/gravimetric energy density. Although the adsorption-based materials demonstrate an enhanced cell performance, they do not provide strong chemical interaction with polysulfide species [30]. To compensate for this problem, polar dopants and functional groups have demonstrated a great potential in interlayer selection. The chemical interaction of PS and the interlayer have been significantly enhanced by incorporating oxygen, nitrogen, and fluorine moieties onto the separator surface [31,32,33]. 

In this work, a fluorinated multi-walled carbon nanotubes (F-MWCNTs) coating is applied to a polypropylene Celgard separator as a modified interlayer to minimize polysulfide shuttle. MWCNTs were fluorinated with different fluorine concentrations using a gas fluorination process. To capture and repel PSs at the cathode/separator interface, fluorination of MWCNT produces highly polar sites on their surface. Additionally, carbon nanotubes’ high electron conductivity enhances charge transfer at the cathode, producing a remarkable high-rate performance. Using traditional electrochemical characterization techniques coupled with theoretical estimations, both the effects of fluorination and fluorine content on the MWCNT network on reducing shuttle behavior in a Li-S cell are investigated. We elucidate that the fluorinated MWCNT enhanced the function of a separator to mitigate polysulfide shuttle effect, and high conductivity of carbon nanotubes increased the reaction kinetics, showing excellent performance at high current.

## 2. Experimental

### 2.1. Preparation of F-MWCNT

F-MWCNT samples were synthesized by direct gas fluorination process under various temperatures and fluorination times using the methodology described elsewhere [34,35]. In a typical experiment, high quality industrial grade MWCNTs (Nanocyl, NC7000) were placed into a Monel boat and loaded into a custom-built fluorination apparatus. The chamber was purged with the flow of helium while the temperature was ramped to 160 °C. At this temperature, the fluorination was carried out for 3 h by passing a 10% fluorine—90% helium gas mixture over the MWCNT powder in the chamber. The unreacted F_2_ gas was absorbed into a trap containing a concentrated water solution of KOH. After cooling the reactor to room temperature, F-MWCNTs were produced. This process resulted in covalent addition of F to MWCNT surface.

F-MWCNT samples with two fluorination levels, 15%, and 27% atomic percentages of fluorine, were obtained by 3 h fluorination of MWCNT at 160 °C and 200 °C. They were designated as FMWCNT-15 and FMWCNT-27, respectively.

### 2.2. Modification of Polypropylene Celgard Separator

Separator coating slurry was made by thorough mixing of F-MWCNT or MWCNT, and polyvinylidene difluoride (PVDF) (9:1 by weight) in N-Methyl-2-Pyrrolidone (NMP). Slurry was coated on a Celgard sheet by a Doctor Blade Atomic Film Applicator at a thickness of 90 µm and a speed of 50 mm/s. Coated Celgard sheet was dried overnight at 60 °C under vacuum and punched into a 16 mm diameter disks. An average loading of 0.15 mg/cm^2^ was obtained for F-MWCNT and MWCNT on the separator.

### 2.3. Electrochemical Cell Assembly

A 7:3 by weight sulfur-graphene composite was mixed with PVDF in a ratio of 9:1 in NMP and coated onto stainless steel disks followed by overnight drying at 60 °C under vacuum. Three Li-S coin cells each were assembled with pristine Celgard and Celgard separators coated with FMWCNT-27, FMWCNT-15, and MWCNT. 60 µL of 1 M LiTFSI {Lithium bis(trifluoromethanesulfonyl)imide} and 0.2 M LiNO_3_ in DOL:DME ether electrolyte, Li metal as the reference and the counter electrode, and S/C cathode were utilized in a CR2032 coin cells purchased from MTI Corporation. The cells were assembled in an argon-filled glovebox with O_2_ and moisture < 0.1 ppm. Galvanostatic charge discharge studies were performed using LAND Battery Testing System. Cyclic voltammetry with a scan rate of 0.1 mV/s, and electrochemical impedance spectroscopic studies were performed using AUTOLAB PGSTAT 302 N ECOCHEMIE. All electrochemical experiments were conducted at room temperature.

### 2.4. Characterizations

Surface morphology of pristine Celgard and separators coated with MWCNT, FMWCNT-15, and FMWCNT-27 were analyzed using a scanning electron microscope (SEM, JSM-6500F, JEOL USA). Crystallographic structures of MWCNT, FMWCNT-15, and FMWCNT-27 were determined using a Rigaku Ultima II X-ray Powder Diffraction system with Cu Kα radiation at λ = 0.154 nm and were analyzed using PDXL Integrated XRD Software Version 2.8.4.0. The X-ray photoelectron spectroscopy (XPS) analysis was carried out using PHI Quantera XPS instrument with a 5 × 10^−9^ Torr chamber pressure and an Al cathode as an X-ray source. The source power was set at 100 W, and pass energies were 26 eV for the core level scans. Atomic-resolution images of FMWCNT-27 sample were analyzed on a FEI Titan Themis 3 S/TEM. Fourier-transform infrared spectroscopy (FTIR) analysis was conducted on a Nicolet 6700 FTIR spectrometer.

### 2.5. Theoretical Calculations

For all studied systems (see the results and discussion section), preliminary geometry optimization was performed via the PM7 Hartree-Fock based semiempirical method [36]. Then, the structures were reoptimized with Density Functional Theory (DFT) method using a 6-21G(d) basis set functions and the Becke three-parameter Lee-Yang-Parr exchange-correlation functional (B3LYP) [37,38]. In DFT simulations, D3 dispersion corrections were applied for van der Waals interactions (DFT-D3) [39]. To reduce the time required for full geometry optimization with DFT, a semi-empirical method that leverages pre-optimized geometry was used to provide reasonable structures in less time. All theoretical simulations were performed with MOPAC2016 and GAUSSIAN09s software.

## 3. Results and Discussion

**Figure 1:**
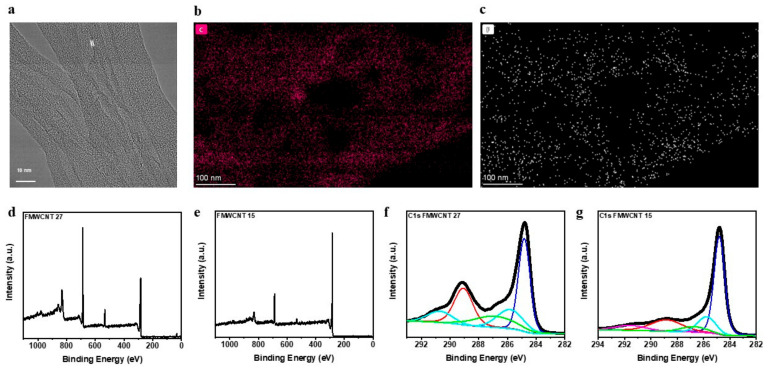
(**a**) High resolution transmission electron microscopic image of FMWCNT-27 sample. TEM-EDS elemental mapping of (**b**) carbon and (**c**) fluorine. X-ray photoelectron spectroscopic survey scans of (**d**) FMWCNT-27, (**e**) FMWCNT-15, and the deconvoluted C1s spectra of (**f**) FMWCNT-27 and (**g**) FMWCNT-15.

To investigate the impact of fluorination on the structure of carbon nanotubes, the sample FMWCNT-27 was analyzed using transmission electron microscopy as shown in the Figure 1a. FMWCNT-27 nanotubes show a diameter ranging from 15–20 nm (Figure S1a). It should be noted that these fluorinated carbon nanotubes retain their shape and show a morphology very similar to pristine MWCNTs. The MWCNTs show graphitic fringes due to hexagonal arrangement of carbon atoms in a tubular shape. Upon fluorination, the FMWCNT-27 sample depicts a multiphase system where the core shows graphitic fringes, which demonstrates that mild fluorination preserves the inherent graphitic structure of MWCNT. At the surface, an amorphous phase is observed (Figure S1b), which can be attributed to intercalation of fluorine atoms in between the rolled graphitic layers [40]. EDS was utilized to understand the distribution of fluorine on FMWCNT-27 in the spectral region depicted in Figure S1c. As seen from Figure 1b,c, the FMWCNT-27 samples show a uniform distribution of fluorine on the surface of MWCNTs.

To understand the bonding between fluorine and the carbon atoms relating to the extent of fluorination, F-MWCNT samples were analyzed using X-ray photoelectron spectroscopy. The survey scans of both FMWCNT-27 and FMWCNT-15 sample indicate the presence of fluorine, carbon, and a small amount of oxygen (Figure 1d,e). The deconvoluted C1s spectra of the FMWCNT-27 and FMWCNT-15 detected six bands (Figure 1f,g). For all the samples, the first significant band was seen at 284.8 eV, which corresponds to sp^2^ bonded carbon atoms in carbon nanotubes. The peak at 286 eV was attributed to the presence of oxygen impurities. The next two peaks observed around 287 eV and 289 eV are attributed to the carbon atoms at the ß-position adjacent to the fluorinated carbon atom and -C-F bonds formed upon fluorination, respectively [40,41]. Deconvoluted fluorine (F1s) spectra display two bands at 688 eV and around 686 eV corresponding to the presence of -F-C bonds on modified MWCNTs and adsorbed fluorine, respectively (Figure S2). Moreover, an increase in fluorine content is demonstrated by an increase in the band at 289 eV of the C1s peak. The sample with a higher fluorine content indicated a 27% surface fluorine concentration while the sample with lower degree of fluorination demonstrated an elemental composition around 14.9% (Table S1).

**Figure 2:**
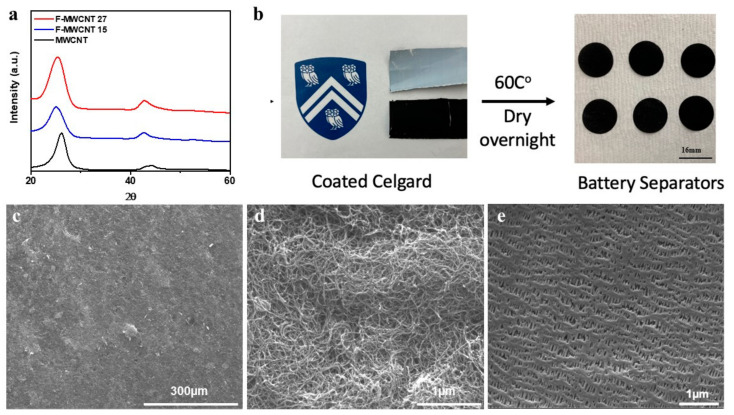
(**a**) X-ray diffraction spectra of pristine MWCNTs (black), FMWCNT-15 (blue) and FMWCNT-27 (red). (**b**) Pictorial representation of the front and back side of a coated separator. After drying, the bifunctional separators were cut into 16 mm diameter disks. SEM images of the coated (**c**,**d**) and opposite side (**e**) of the FMWCNT-27 bifunctional separator.

X-ray diffraction was used to analyze the structural properties of MWCNT and F-MWCNT; the obtained diffraction patterns are shown in Figure 2a. The first significant peak for MWCNT, known as (002) planes, is visible at about 26°. This broad feature can be identified with the characteristic graphitic peak appearing due to the tubular structure of carbon in the MWCNT sample [42]. For F-MWCNT-15 and F-MWCNT-27, the XRD peaks shifted to approximately 25.0° and 42.5°, indicating an increase in the interplanar distance after fluorination. Additionally, the relative intensity of the broad band at around 25.0° decreased in the XRD of both F-MWCNT-15 and F-MWNCT-27, following the trend noted in the literature [40].

The modified MWCNTs were uniformly coated on a pristine Celgard separator by making a (9:1) slurry with PVDF binder) in NMP solvent and dried overnight at 60 °C. Scanning Electron Microscopy (SEM) was used to examine the morphology of the coated separator. Lower magnification images reveal a homogeneous distribution of the fluorinated MWCNTs on the Celgard 2500 separator (Figure 2c). Higher magnification images (Figure 2d) show that the F-MWCNTs create a homogeneous porosity network on the surface that serves as both a “second current collector” and a pathway for ions to move through. A major concern for the bifunctional separators is the impregnation of conducting materials through the separator. SEM image taken on the opposite side of the coated separator showed that the MWCNTs does not seep through the separator (Figure 2e).

**Figure 3:**
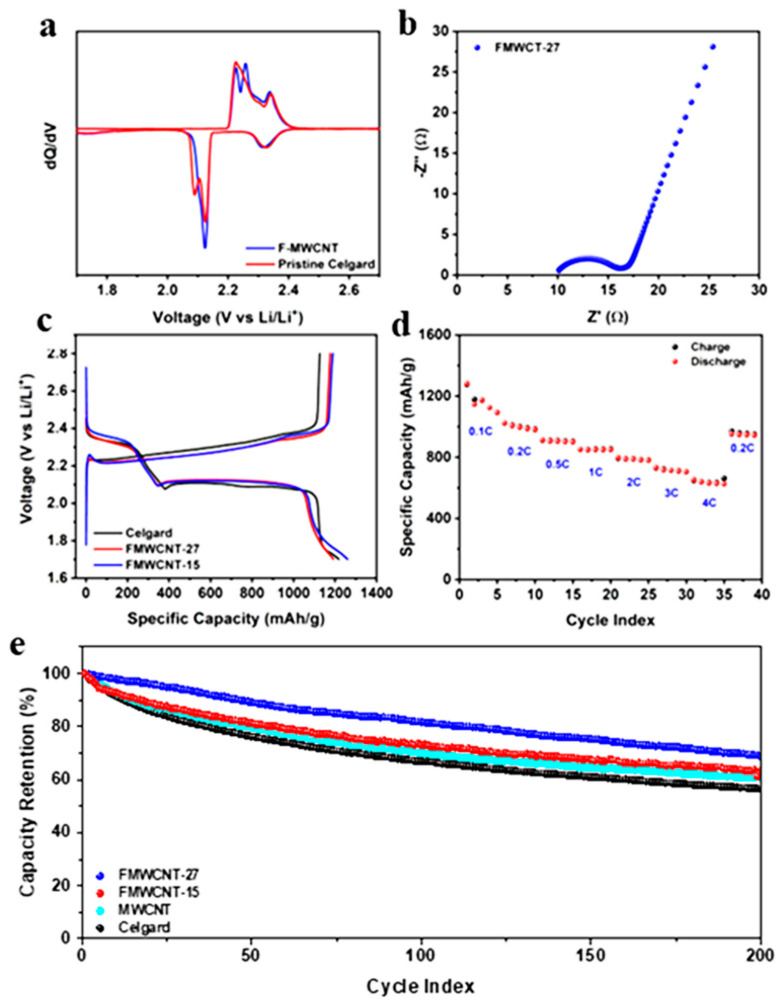
(**a**) Differential capacity plots for FMWCNT-27 and pristine Celgard and (**b**) electrochemical impedance spectroscopy data of the FWCNT-27 coated separator. (**c**) First cycle charge–discharge profiles for cells with pristine Celgard separator (black), FWCNT-15 (blue), FMWCNT-27 (red) coated separator at 0.1 C, and (**d**) rate performance charts for FMWCNT-27 from 0.1 C to 4 C. (**e**) Capacity retention for cells with pristine Celgard (black), FWCNT-15 (red) and FMWCNT-27 (blue) coated separator tested at 1 C.

Figure 3a displays the first cycle differential capacity curves for the cells withFMWCNT-27 and pristine Celgard. The cells with pristine Celgard separator show three peaks during the discharge. Whereas, for the cells with modified separator, two distinct discharge peaks are visible, suggesting improved kinetics due the modified separator [43,44]. Additionally, small intrinsic resistance (R_s_) of 10 Ω and a very small charge transfer impedance (R_ct_) of about 6.5 Ω were measured from the Li-S battery with the modified separator, further confirming the efficient electrochemical nature of this electrode (Figure 3b). This value, when compared to previous reports, is exceptional as charge transfer resistances are usually measured between 15 Ω and 100 Ω, or even greater [10,45,46].

First cycle charge-discharge profile for the four cells is shown in Figure 3d. All the cells depicted a typical Li-S galvanostatic charge-discharge (GCD) curves with the first discharge plateau occurring between 2.3–2.4 V and the second discharge plateau around 2.1 V; however, the second discharge plateau of the cell with pristine Celgard occurred at a lower potential than cells with a modified separator. This trend was also detected in the charge plateaus, where the first plateau appeared at a higher potential in the cells with pristine Celgard. The difference in potential of the charge and discharge plateaus was inversely proportional to the charge-transfer resistance, suggesting a better conductivity in the cells with modified separators. This observation supported the small charge transfer impedance and intrinsic resistance from the impedance spectroscopy data mentioned previously. This finding indicates that incorporating MWCNTs at the interface improved charge-transfer resistance, thus the F-MWCNT/Celgard system should be a promising candidate for high-rate applications.

GCD studies performed at different current rates ranging from 0.1 C to 4 C to understand the rate performance of the FMWCNT-27 coated Celgard separator and the corresponding curves are shown in Figure 3d. During the first five cycles at 0.1 C, the capacity faded to 1142 mAh g^−1^, while the cell retained 1030 mAh g^−1^ at 0.2 C, 950 mAh g^−1^ at 0.5 C and 900 mAh g^−1^ at 1 C. However, with the ever-increasing demand for fast charging in current electronic devices, lithium secondary batteries require charging time of less than an hour. Due to the presence of F-MWCNT on the separator, the Li-S system demonstrated an exceptionally high-rate performance with discharge capacities of 830 mAh g^−1^ at 2 C, 754 mAh g^−1^ at 3 C and 676 mAh g^−1^ at 4 C (Figure 3d). These results indicated that the cell can be charged in 15 min while providing 40% theoretical capacity of a Li-S system.

Delving into the mechanism of the improved electrochemical performance, an introduction of fluorine by bonding to the MWCNTs increases the electron density on the surface of the modified functional membrane. Abundant lone pairs around fluorine atoms generate a high electron density on the surface of MWCNT to continuously repel the shuttling polysulfides, while MWCNT further ensures the entrapment of the remaining polysulfides inside of the porous network structure. The difference in potential caused by MWCNT fluorination drives polysulfides to mitigate through porous separators, which allows the FMWCNT coating to (1) inhibit the migration of intermediate species by creating a polar interlayer; (2) reduce the charge-transfer resistance of the cell at the cathode interface as observed from the EIS data; (3) increase sulfur utilization at the cathode by utilizing CNTs as the second current collector and; and (4) provide mechanical stability for volumetric expansion of sulfur upon lithiation. An improvement in capacity retention was detected when the Li-S cells with modified Celgard separators were cycled with different concentrations of fluorine in the F-MWCNTs using GDC and the results were compared with the pristine Celgard separator and MWCNT coated Celgard separator. All the cells were rested for 12 h prior to cycling, cycled at 0.1 C for two cycles to stabilize the reaction kinetics, and tested at 1 C for the remaining number of cycles. The cells with pristine Celgard showed a capacity of around 1200 mAh g^−1^ in the first cycle, achieving a capacity of 410 mAh g^−1^ after 200 cycles. The cells with Celgard coated with pristine MWCNT show a capacity of around 450 mAh g^−1^ while the FMWCNT-27 and FMWCNT-15 coated separators retained a higher value of 610 mAh g^−1^ and 590 mAh g^−1^, respectively, after 200 cycles (Figure S3). However, a clearer understanding of the effect of fluorine on the MWCNTs could be obtained only by comparing the capacity retention after a given number of cycles. To further investigate the effect of fluorination on cycling performance, percentage capacity retention of each cell was calculated with respect to the third cycle capacity or 1st cycle at 1C. As seen in Figure 3e, Celgard separator, which had no coated carbon surfaces, shows maximum capacity retention of around 56% after 200 cycles. Higher capacity retention was attained when FWMCNT-15 and MWCNT were added to the separator, with FMWCNT-15-containing cells performing better (64% retention) than MWCNT-containing cells (60% retention). A sharp increase in the capacity retention was observed when cells with higher fluorine content (FMWCNT-27) were utilized. Utilizing cells with a greater fluorine content (FMWCNT-27) led to the significant capacity retention increase. The cells that contained FMWCNT-27 had the best capacity retention after 200 cycles at 1 C, at about 70%. The initial idea that fluorinated MWCNT played a crucial role in efficiently capturing and repelling PSs at the cathode/separator interface was substantiated by its higher capacity retention.

The behavior of the lithium polysulfides in contact with the fluorinated CNTs was examined theoretically to simulate the impacts of F-MWCNTs on battery performance. The results showed that using the fluorinated MWCNT can resist polysulfides movement and cause lithium release. PM7 and DFT-D3 electronic structure simulations were carried out (as described in Experimental section) to evaluate the interaction between the Li_2_S_4_ and Li_2_S_8_ in contact with a single-walled (6, 6) CNT, 20 Å long (~216 atoms), and three similar structures with fluorination levels of 15%, 21%, and 27% with fluorine atoms randomly distributed along the CNT. The ends of CNTs were passivated with hydrogen atoms. Li_2_S_4_ and Li_2_S_8_ were chosen because the most soluble lithium sulfides, containing from four to eight sulfur atoms, are the most likely to interact with the battery separator among other Li_2_S_n_ [47]. Previous study confirmed that the property of CNT’s surface is a crucial factor to consider in regard to the interactions between PS and the fluorinated CNTs by comparing the PS repulsion of MWCNT and double-walled CNTs. Based on that conclusion, the theoretical simulation study below will be restricted to discuss the single-walled CNT cases.

**Figure 4:**
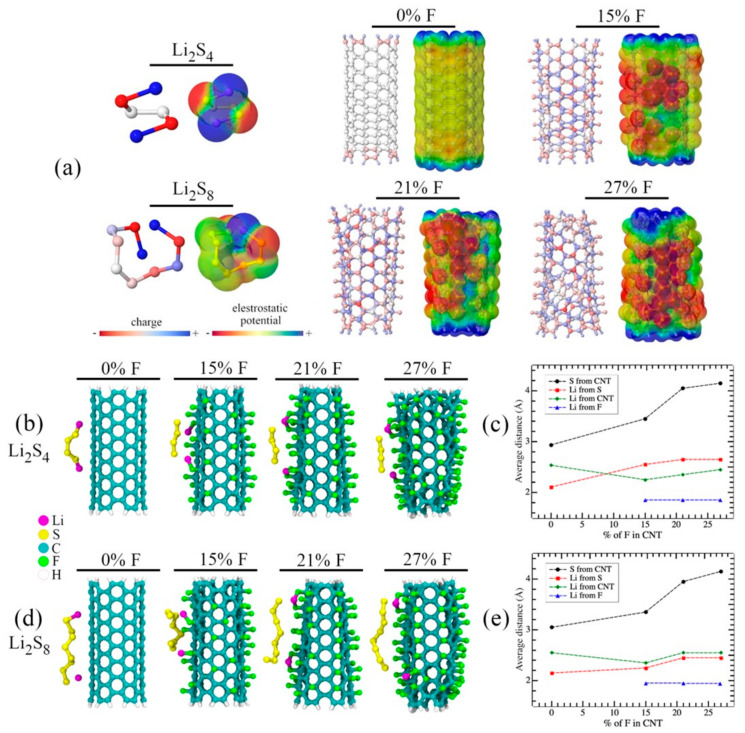
(**a**) Charge distribution and the electrostatic potential surface of the studied systems after geometry optimization. (**b**) Li_2_S_4_ in contact with the pristine and fluorinated CNT and (**c**) the average distance between each constituent as a function of the percentage of fluorination. (**d**,**e**) The corresponding ones for Li_2_S_8_ in contact with the pristine and fluorinated CNT.

Figure 4 presents the overall results from the simulations. Figure 4a shows the partial charge distribution (estimated from Mulliken population analysis) and the corresponding electrostatic potential surface of the studied systems after the geometric optimization. The red to blue color scale for the charge distribution are designated as the more negative (excess of electrons) and more positive (absence of electrons) regions, respectively. For electrostatic potential surface, the red to blue color scale represents the most attractive and repulsive regions, respectively. The electrostatic potential surface reflects that the negative charges from the lithium polysulfides are more concentrated on the sulfur terminations due to the attraction of lithium atoms. For larger lithium polysulfide (Li_2_S_8_), sulfur atoms experienced a more positive charge distribution. For the CNTs, it is evident that the fluorination process induces structural distortions proportional to the fluorination level. In the pristine CNTs, the electronic charge is evenly distributed. However, our analysis results showed that the charge distribution on the fluorinated CNTs became less regular. Due to the more electronegative fluorine atoms, some carbon atoms became more positive than others. From the electrostatic potential surface, the insertion of the fluorine atoms was observed to induce reactivity on the CNT surface, in which some very attractive regions around the fluorine atoms were formed to modify the interaction with the lithium PS compared to pristine CNTs.

Lithium PS repulsion effect was further investigated when placing PS near pristine CNT and fluorinated CNTs. To undertake a geometry optimization of the complete PS-CNT system and assess the final positions of each ingredient, Li_2_S_4_ and Li_2_S_8_ were placed 2.5 Å apart from the CNTs; the simulation results are shown in Figure 4b–e. Figure 4b depicts the structures after the geometry optimization of the Li_2_S_4_ in contact with the pristine CNT and the fluorinated CNT. Figure 4c shows the average distance between S-CNT, Li-S, Li-CNT, and Li-F of the Li_2_S_4_ in contact with the pristine and fluorinated CNT after the geometry optimizations. When compared to the pristine CNTs, it was found that the fluorinated CNTs typically attracted more Li atoms and kept the sulfide chain farther away. Li atoms became more distant from the polysulfide, with a distance increasing from 2.1 Å to 2.6 Å measuring from the terminal sulfur atoms as the fluorination level increases, suggesting that Li atoms prefer to be closer to the fluorine atoms (the most attractive regions) than to remain attached to the polysulfides. The estimated binding energies (BE) are 1.36, 4.74, 5.14, and 5.20 eV for Li_2_S_4_ in contact with the pristine and 0%, 15%, 21%, and 27% fluorinated CNTs, respectively.

These results indicated that the fluorine atoms in CNTs increased the interaction with lithium PS. It is important to note that the estimated BE was larger than that reported for lithium polysulfides in contact with CNTs functionalized with OH and amino groups [48,49], proving that fluorinated CNTs confined the lithium PS in the cathode side more efficiently.

Figure 4d,e present the corresponding data for Li_2_S_8_ in contact with the pristine and fluorinated CNTs. Consistent with the obtained results for Li_2_S_4_, the sulfide chain maintained more distant, and the Li atoms became more attracted to the CNT with an increasing fluorination rate. As shown in Figure 4e, the sulfur atoms of Li_2_S_8_ were slightly closer to the CNTs compared to Li_2_S_4_ because Li_2_S_8_ has an increased degree of freedom in relation to Li_2_S_4_. In Figure 4a, some sulfur atoms were more positive, inducing a stronger interaction with the negative cloud in the CNT. The estimated binding energies (BE) were found as 1.58, 5.04, 5.27, and 5.76 eV for Li_2_S_8_ in contact with 0%, 15%, 21%, and 27% fluorinated CNTs, respectively. These values were slightly greater than the corresponding ones for the Li_2_S_4_, confirming that fluorination improved the interaction between CNTs and lithium PS.

Theoretical modeling findings showed that fluorination caused the release of lithium atoms and successfully repelled the sulfides from the CNTs. The benefits of these combined actions include preventing PS diffusion towards the anode, allowing the recycling of PS that are maintained on the cathode side, and extending the lifespan of Li-S batteries. The fluorine interlayer’s better capacity retention suggests that highly polar carbon additions are a potential new way to improve Li-S batteries. However, given that energy density is an important matrix in determining the performance of Li-ion batteries, a great deal of research is needed to understand the effective loading of these lightweight, polar, and conducting interlayers.

## 4. Conclusions

A uniform coating of fluorinated MWCNTs on the separator traps and repels the polysulfides due to a high electron density of the fluorine atoms and the network structure of MWCNT. The study was conducted by varying the atomic percentage of fluorine in the F-MWCNTs and monitoring its effect on capacity retention over 200 cycles. The presence of fluorine interlayer shows no parasitic reactions in the Li-S cells. Furthermore, compared to cells with a traditional separator and cells coated with MWCNTs, the Li-S battery with a 27% atomic fluorine concentration in the interlayer showed the best capacity retention. Due to the excellent electronic conductivity of carbon nanotubes, the charge transfer resistance at the cathode is greatly reduced. This was further supported by an improved rate-performance with a specific discharge capacity around 676 mAh g^−1^ at 4 C. Overall, our findings suggested that the use of a F-MWCNT-modified separator in a Li-S cell is a promising solution to the problem of not only polysulfide shuttle but also the high-rate requirement in current electronics.

## Data Availability

Additional data are available in the Supplementary Material.

## Supplementary Materials

The following supporting information can be downloaded at: https://www.mdpi.com/article/10.3390/ma16051804/s1.
