# Supplementary material for: Fluorinated Multi-Walled Carbon Nanotubes Coated Separator Mitigates Polysulfide Shuttle in Lithium-Sulfur Batteries
Source: Materials (Basel). 2023 Feb 22;16(5):1804. doi: 10.3390/ma16051804 (PMC10004040; doi:10.3390/ma16051804)
Supplement: Supplementary file 1 [file materials-16-01804-s001.zip › materials-2119264-supplementary.pdf]

SUPPLEMENTARY INFORMATION

Fluorinated multi-walled carbon nanotubes coated separator mitigates polysulfide shuttle in lithium-sulfur batteries

Devashish Salpekar[1,¶,], Changxin Dong[1,¶], Eliezer F. Oliveira[1,2,3], Valery N. Khabasheskul[1,*], Guanhui Gao[1], Ved Ojha[1], Robert Vajtai[1], Douglas S. Galvao[2,3], Ganguli Babu[1,*] and Pulickel M. Ajayan[1,*]*

[1] Department of Material Science and NanoEngineering, Rice University, Houston, Texas, 77005, USA.

[2] Group of Organic Solids and New Materials, Gleb Wataghin Institute of Physics, University of Campinas (UNICAMP), Campinas, SP, Brazil.

[3] Center for Computational Engineering & Sciences (CCES), University of Campinas (UNICAMP), Campinas, SP, Brazil.

[¶] These authors contributed equally to this work

*Email: salpekardevashish@gmail.com; khval@rice.edu; babu.ganguli@rice.edu; ajayan@rice.edu

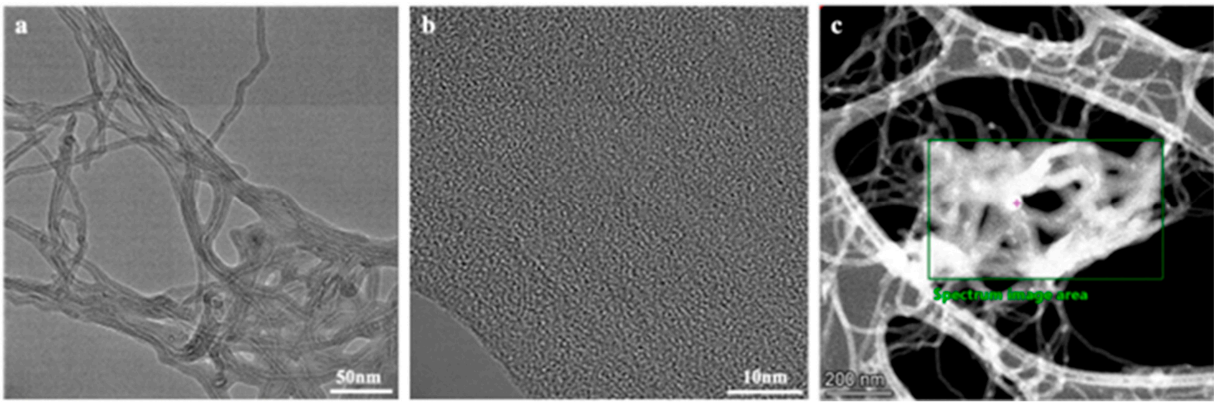

Figure S1. (a) Low resolution TEM image of the fluorinated MWCNTs. (b) HRTEM image of FMWCNT-27 clearly depicting the multi-phase system. The core graphitic structure of the CNTs is surrounded by an amorphous phase attributed to the fluorine intercalation in the outer walls of CNTs. (c) TEM HAADF image of the spectral area analyzed using EDS.

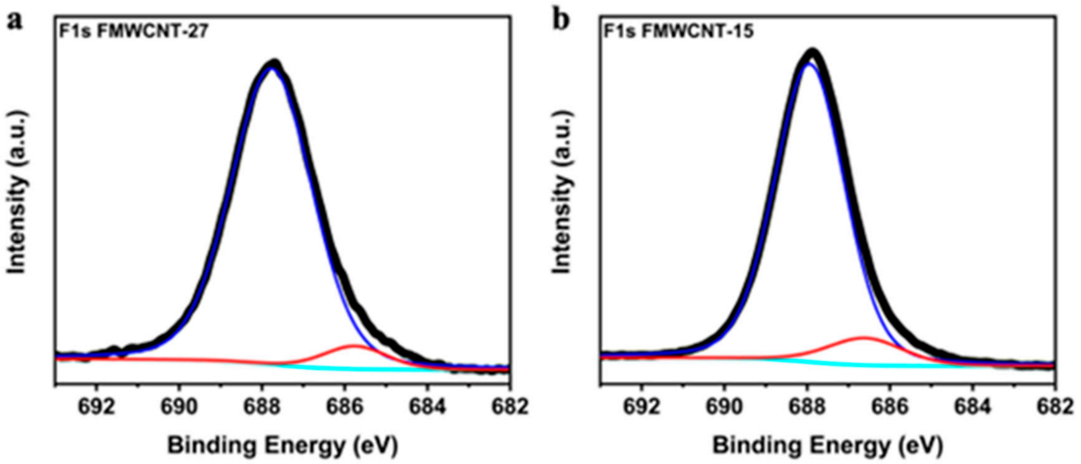

Figure S2. Deconvoluted F1s spectra of (a) FMWCNT-27 and (b) FMWCNT-15.

Table S1. Elemental Composition (at.%) of the two fluorinated MWCNTs obtained using XPS

**Sample**	**% C**	**% F**	**% O**
**FWMCNT-15**	84.2	14.9	0.9
**FMWCNT-27**	70.9	26.8	2.3

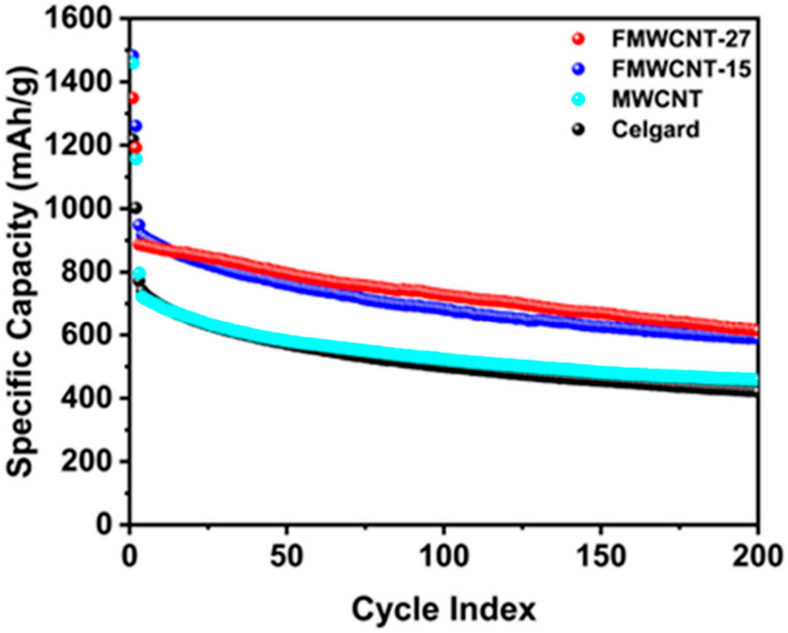

Figure S3. Charge-discharge cycles Li-S cells with FMWCNT-27 (red), FMWCNT-15 (blue) and MWCNT (cyan) coated Celgard separator compared to Li-S cell with Pristine Celgard separator (black) tested at 1C.
